# Cardiac thrombi detected by CT in patients with acute ischemic stroke: A substudy of Mind the Heart

**DOI:** 10.1177/23969873221130838

**Published:** 2022-10-26

**Authors:** Leon A Rinkel, Chiel FP Beemsterboer, Nina-Suzanne Groeneveld, Nick HJ Lobé, S Matthijs Boekholdt, Berto J Bouma, Fenna F Muller, Ludo FM Beenen, Henk A Marquering, Charles BLM Majoie, Yvo BWEM Roos, Adrienne van Randen, R Nils Planken, Jonathan M Coutinho

**Affiliations:** 1 Department of Neurology, Amsterdam UMC, University of Amsterdam, Amsterdam, The Netherlands; 2 Departments of Radiology and Nuclear Medicine, Amsterdam UMC, University of Amsterdam, Amsterdam, The Netherlands; 3 Department of Cardiology, Amsterdam UMC, University of Amsterdam, Amsterdam, The Netherlands; 4 Departments of Biomedical Engineering and Physics, Amsterdam UMC, University of Amsterdam, Amsterdam, The Netherlands

**Keywords:** Acute stroke, cardiac emboli, computed tomography angiography

## Abstract

**Background:**

Cardiac thrombi are a major risk factor for ischemic stroke, but are rarely diagnosed in the acute phase. We examined characteristics and functional outcome of patients with ischemic stroke and a concomitant cardiac thrombus detected on cardiac CT performed in the acute phase.

**Patients and Methods:**

We used data from “Mind the Heart,” a prospective cohort study in which consecutive adult patients with acute ischemic stroke underwent prospective ECG-gated cardiac CT during their acute stroke imaging protocol. We compared characteristics, functional outcome (modified Rankin scale) and stroke recurrence rate at 90 days of patients with a cardiac thrombus on CT (defined as filling defect <100 Hounsfield Units) to those without a cardiac thrombus.

**Results:**

Among 452 included patients, cardiac CT detected 41 thrombi in 38 (8%) patients. Thrombi were most often located in the left atrial appendage (31/38 [82%]). Patients with a cardiac thrombus more frequently had intracranial occlusions in multiple vascular territories (5% vs 0.5%, *p* = 0.04) and a higher baseline NIHSS score (17 [IQR 6–22] vs 5 [IQR 2–3], *p* < 0.001) compared to patients without a cardiac thrombus. In 13/38 (34%) patients with a cardiac thrombus, no atrial fibrillation was detected. A cardiac thrombus was associated with worse functional outcome (adjusted common odds ratio 3.18 95%CI 1.68–6.00). Recurrence rate was not significantly different (8% vs 4%, aOR 1.50 (0.39–5.82).

**Discussion and Conclusion:**

Cardiac CT detected a cardiac thrombus in one in every 12 patients with acute ischemic stroke, and these patients had more severe deficits, multivessel occlusions, and a worse functional outcome.

## Graphical abstract

**Graphical abstract:**
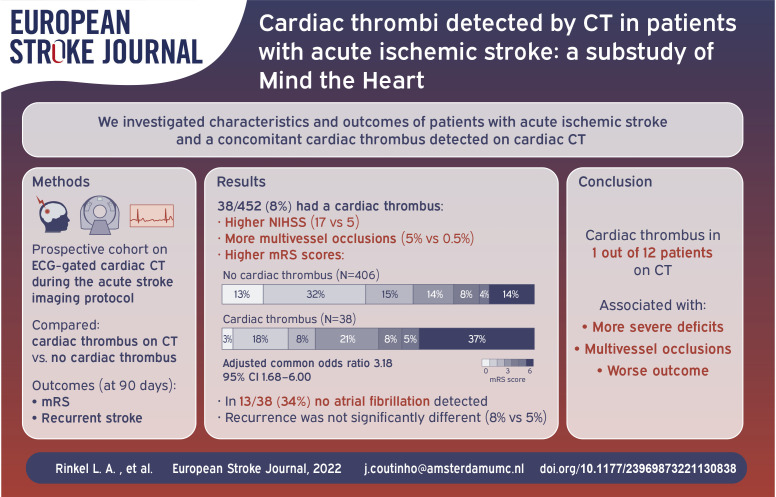

## Introduction

Cardiac thrombi are a major risk factor for acute ischemic stroke and are most frequently found in the left atrial appendage or left ventricle.^1–3^ The diagnosis of a cardiac thrombus will generally lead to the consideration of anticoagulation, even in the setting of acute ischemic stroke. In addition, long-term anticoagulation may be indicated in ischemic stroke patients who have a cardiac thrombus, even without concurrent atrial fibrillation. Transthoracic echocardiography (TTE) is the most widely used modality to screen for structural sources of cardio embolism in patients with stroke,^4^ but TTE has a low diagnostic yield for thrombi due to its limited capability to visualize the left atrial appendage.^5^ Transesophageal echocardiography (TEE) has a higher diagnostic yield but is invasive and may require sedation, and is therefore not routinely performed in all stroke patients.^4^ Moreover, echocardiography is often delayed for several days or even weeks, at which time cardiac thrombi may no longer be visible.^6–8^

Cardiac CT acquired during the acute stroke imaging protocol is a suitable first-line screening method to detect cardiac thrombi. The diagnostic yield of CT for cardiac thrombi ranges from 10%–24% in cohort studies,^9–13^ and is higher compared to TTE.^14^ Previous studies have described worse outcome after stroke in patients with atrial fibrillation-related stroke,^15,16^ but there is a paucity of studies reporting on functional outcome in ischemic stroke patients with a cardiac thrombus. We aimed to compare characteristics, functional outcome and risk of recurrent stroke in ischemic stroke patients with and without a cardiac thrombus detected on cardiac CT.

## Methods

This study is a sub study of the Mind the Heart study.^14,17^ Mind the Heart was a prospective single-center cohort study performed in Amsterdam UMC, a comprehensive stroke center in the Netherlands. The aim of the study was to determine whether cardiac CT acquired in the acute phase of stroke has a higher diagnostic yield for structural sources of embolism compared to transthoracic echocardiography and may therefore be a suitable screening method in patients with ischemic stroke. Between 2018 and 2020, consecutive adult patients with acute ischemic stroke who were potentially eligible for reperfusion therapy (intravenous thrombolysis [IVT] or endovascular treatment [EVT]) at the time of admission (i.e. patients with acute onset neurological symptoms that developed less than 24 h ago) were included. Patients underwent prospective ECG-gated cardiac CT during the initial stroke imaging protocol, immediately following non-contrast-enhanced CT of the brain, CT perfusion, and non-gated CT-angiography of the aortic arch, cervical and intracranial arteries, resulting in an additional median scan time of 6 min and a median door-to-cardiac CT time of 32 min. Patients also underwent routine stroke work-up, including TTE. TEE was not performed routinely. Additional details of the study have been published.^14^

For the current study, we compared patients with a cardiac thrombus on CT to those without a cardiac thrombus. Cardiac thrombus on CT was defined as a left atrial, left atrial appendage or left ventricular filling defect <100 Hounsfield Units (HU).^18^ Left atrial appendage fillings defects >100 HU were classified as slow-flow.^18^ An intracranial large vessel occlusion was defined as an occlusion in the intracranial internal carotid artery, A1, M1, M2, or basilar artery.

We performed a structured interview by telephone 3 months after the index stroke to determine functional outcome, measured on the modified Rankin Scale (mRS, range 0 [no disability] to 6 [death]) and to collect information on secondary stroke prevention measures and the occurrence of cardiovascular events, including recurrent stroke.

Outcomes of the study were: mRS at 90 days, functional independence (defined as mRS 0–2), mortality and recurrent ischemic stroke at 90 days.

### Statistical analysis

We compared patients with a cardiac thrombus on CT with patients without a cardiac thrombus. Differences in characteristics, including acute stroke treatment, between patients with versus without a cardiac thrombus were assessed using Mann-Whitney U test and chi-square test as appropriate. To assess the association between presence of a cardiac thrombus and functional outcome we used univariable and multivariable ordinal logistic regression to evaluate a shift toward poorer functional outcome on the mRS. The regression analysis was adjusted for the following potential confounders: age, atrial fibrillation, pre-stroke mRS, and anticoagulation use. We performed binary logistic regression adjusted for the same potential confounders to assess the association between cardiac thrombi and the other outcomes. We did not adjust for baseline National Institute of Health Stroke Scale (NIHSS) score and stroke recurrence in these analyses as these variables are most likely on the causal pathway in the association between presence of cardiac thrombus and functional outcome. To determine whether NIHSS score at baseline and ischemic stroke recurrence at 90 days explain the association between cardiac thrombi and functional outcome, we performed a sensitivity analysis in which we added these variables as additional adjustments to the regression model. We imputed missing data for the regression analyses using multiple imputation methods.^19^

Analyses were performed using R software, version 4.0.3 (R foundation for Statistical Computing 2018). We used a significance level of 0.05 for all tests.

## Results

**Figure 1.**
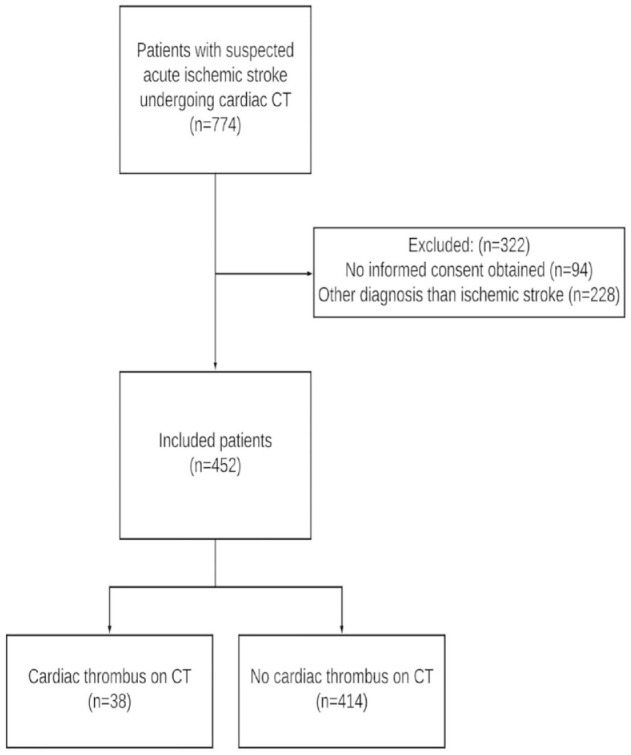
Flowchart of patients.

Of 452 included patients, cardiac CT detected 41 thrombi in 38 (8%) patients (Figure 1). Thrombi were located in the left atrial appendage in 31 (82%), left atrium in two (5%) and left ventricle in seven (18%) patients. The two patients with a left atrium thrombus had a concomitant left atrial appendage thrombus. One patient had two separate thrombi in the left atrial appendage. TTE was acquired in 350 patients and identified a thrombus in only two patients (0.6%), both in the left ventricle.

Median age was 72 (interquartile range [IQR] 62–80) and 76 (IQR 63–87, *p* = 0.13) for patients with and without a cardiac thrombus, respectively. Patients with a cardiac thrombus more frequently had a history of atrial fibrillation (40% vs 15%, *p* < 0.001), intracranial occlusions in multiple vascular territories (5% vs 0.5%, *p* = 0.04), and a higher baseline NIHSS (17 [IQR 6−22] vs 5 [IQR 2−3], *p* < 0.001, Table 1) compared to patients without a cardiac thrombus. In patients with a cardiac thrombus, four out of nine patients (44%) who used a vitamin K antagonist at baseline had an INR < 2.0 compared to 13/27 (48%) in patients without a cardiac thrombus. In the group with a cardiac thrombus, seven (18%) were treated with direct oral anticoagulants (DOAC) at baseline compared to 39 (9%) in patients without a cardiac thrombus (*p* = 0.48). Although rates of treatment with IVT and EVT were numerically higher for patients without a cardiac thrombus, we did not observe a statistically significant difference (IVT: 29% vs 42%, *p* = 0.17, EVT: 18% vs 23%, *p* = 0.66). In the group without a cardiac thrombus, 68 (16%) patients had left atrial appendage slow-flow.

**Table 1.** Baseline characteristics.

	No cardiac thrombus *N* = 414	Cardiac thrombus *N* = 38	*p* value
Age—year	72 (62–80)	76 (63–87)	0.13
Male sex	248 (60)	20 (53)	0.48
Systolic blood pressure—mmHg^a^	152 (135–171)	156 (130–180)	0.50
Diastolic blood pressure—mmHg^a^	86 (76–99)	85 (75–98)	0.73
NIHSS score	5 (2–13)	17 (6–22)	<0.001
Medical history			
Previous ischemic stroke^b^	72 (17)	10 (26)	0.26
Transient ischemic attack^b^	37 (9)	4 (11)	0.98
Atrial fibrillation^b^	62 (15)	15 (40)	<0.001
Diabetes mellitus^b^	66 (16)	6 (16)	1.00
Hypertension	190 (46)	19 (50)	0.75
Hypercholesterolemia	61 (15)	9 (24)	0.22
Smoking^c^	152 (40)	11 (31)	0.36
Malignancy	56 (14)	7 (18)	0.56
Myocardial infarction	48 (12)	11 (29)	<0.01
Chronic heart failure	15 (4)	2 (5)	0.65
Median pre-stroke modified Rankin Scale score^d^	0 (0–1)	0 (0–1)	0.12
Medication use			
Anticoagulation	68 (16)	16 (42)	<0.001
DOAC	39 (9)	7 (18)	0.48
Non-compliance/Discontinuation*	7 (18)	1 (14)	0.29
Vitamin K antagonist	27 (7)	9 (24)	0.33
INR < 2.0^e^	13 (48)	4 (44)	1.00
Antiplatelet	126 (30)	8 (21)	0.31
Anti-hypertensive drugs	212 (51)	27 (71)	0.03
Statin	140 (34)	15 (40)	0.60
Intracranial large vessel occlusion	171 (41)	21 (55)	0.10
Intracranial occlusion in multiple vascular territories	2 (0.5)	2 (5)	0.04
Reperfusion therapy			
IV thrombolysis	173 (42)	11 (29)	0.17
Endovascular thrombectomy	95 (23)	7 (18)	0.66
Process time, median duration			
Onset-to-door time^f^—min	176 (72–555)	142 (50–517)	0.42
Door-to-needle time^g^—min	41 (31–57)	44 (32–73)	0.82
Door-to-groin time^h^—min	66 (53–82)	68 (52–76)	0.94
TOAST classification^i^			
Cardioembolic	108 (26)	35 (92)	<0.001
Larger artery atherosclerosis	47 (11)	0 (0)	
Small vessel disease	55 (13)	0 (0)	
Other determined	30 (7)	0 (0)	
Unknown	174 (42)	3 (0)	
Incomplete evaluation	0	0 (0)	
Negative evaluation	163 (39)	0 (0)	
More than one cause	11 (1)	3 (8)	

Data are reported as median (interquartile range), or as number (percentage), as appropriate.

NIHSS: National Institute of Health Stroke Scale; DOAC: Direct oral anticoagulants.

* Non-compliance in three patients, all in patients without a cardiac thrombus. Temporarily discontinuation for diagnostics/intervention in the remaining patients.

Missing values, n (%): ^a^2 (0.4), ^b^1 (0.2), ^c^35 (8), ^d^10 (2), ^e^3 (8), ^f^45 (10), ^g^6 (4), ^h^2 (2). ^i^Including results of cardiac CT.

Of the 38 patients with a cardiac thrombus detected on CT, 15 (40%) had a previous medical history of atrial fibrillation and 10 (26%) had *de novo* atrial fibrillation detected on baseline electrocardiogram or rhythm monitoring during stroke work-up. Hence, no atrial fibrillation was detected in 13 (34%) patients with a cardiac thrombus. Thrombi in these 13 patients were located in the left atrial appendage (*n* = 7) and left ventricle (*n* = 6). At 90 days, 19/23 (83%) patients with a cardiac thrombus who were alive were treated with anticoagulation. One patient was not treated with anticoagulation because TTE and TEE did not show a cardiac thrombus, in one patient anticoagulation was initiated after the 90-day follow-up period. For the remaining two patients the reason for withholding anticoagulation was not recorded.

**Figure 2.**
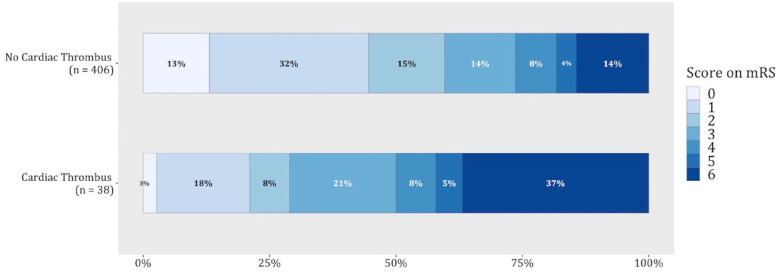
Functional outcome at 90 days.

The full range of mRS scores in patients with versus without a cardiac thrombus is provided in Figure 2. After adjustment, presence of a cardiac thrombus was associated with a worse overall outcome (adjusted common odds ratio for a shift toward poor outcome: 3.18 95% confidence interval [CI] 1.68–6.00, Table 2). Presence of a cardiac thrombus was also associated with lower rates of functional independence (adjusted OR [aOR] 0.28, 95%CI 0.12–0.62) and higher mortality rates (aOR 3.26, 95%CI 1.43–7.43) at 90 days. Patients with a cardiac thrombus had a numerically higher rate of recurrent ischemic stroke than patients without a cardiac thrombus at 90 days (8% vs 4%, aOR 1.50 (0.39–5.82), although this difference was non-significant.

**Table 2.** Outcomes at 90 days.

	No cardiac thrombus *N* = 414	Cardiac thrombus *N* = 38	Unadjusted common odds ratio (95% confidence interval)	Adjusted common odds ratio (95% confidence interval)
Median mRS^a,b^	2 (1–4)	4 (2-6)	3.33 (1.82–6.07)	3.18 (1.68–6.00)
Functional independence (mRS 0–2)^b^	242/406 (60%)	11/38 (29%)	0.27 (0.13–0.56)	0.28 (0.12–0.62)
Mortality^b^	58/406 (14%)	14/38 (37%)	3.55 (1.73–7.28)	3.26 (1.43–7.43)
Ischemic stroke recurrence^c^	17/396 (4%)	3/38 (8%)	2.00 (0.56–7.19)	1.50 (0.39–5.82)

mRS denotes modified Rankin Scale.

Regression analyses were adjusted for the following potential confounders: age, atrial fibrillation, pre-stroke mRS and anticoagulation use.

a Sensitivity analysis additionally adjusted for NIHSS and ischemic stroke recurrence at 90 days: adjusted common odds ratio: 2.24, 95% confidence interval 1.18–4.27.

Missing values, n (%): ^b^8 (2), ^c^18 (4).

The sensitivity analysis showed that the association between presence of a cardiac thrombus and worse functional outcome became less pronounced but was still present after adjusting for baseline NIHSS score and ischemic stroke recurrence (adjusted common odds ratio for a shift toward poor outcome: 2.22 95% confidence interval [CI] 1.16–4.23)

## Discussion

In this prospective cohort study including patients with acute ischemic stroke, cardiac CT detected a cardiac thrombus in one in every 12 patients. These patients more often had atrial fibrillation, severe neurological deficits and more often had a stroke in multiple vascular territories. The presence of a cardiac thrombus was also associated with a worse functional outcome. While the risk of stroke recurrence was numerically higher in patients with a cardiac thrombus, this difference was not statistically significant.

Previous studies have reported an association between atrial fibrillation-related stroke and worse functional outcome.^15,16^ Our findings suggest that this association may have been driven by the worse clinical outcome in patients with undetected cardiac thrombi. The association between cardiac thrombi and poor outcome may be mediated by a combination of more severe strokes due larger thrombi causing more proximal occlusions, a higher rate of multivessel occlusions, less use of IVT due to anticoagulation therapy, and a higher short-term ischemic stroke recurrence rate. Indeed, we observed that patients with cardiac thrombus more often had higher NIHSS scores, multivessel occlusion and that the rates of IVT treatment were numerically lower for these patients. We found that the association between presence of a cardiac thrombus and functional outcome became less strong after adjusting for baseline NIHSS and stroke recurrence, but still persisted. This indicates that these variables only partially explain the observed association. However, this finding should be considered as hypothesis generating only. Larger studies are needed to confirm the association between a cardiac thrombus and functional outcome, and to explore whether this association is mediated by pathophysiological mechanisms caused by cardiac thrombi or whether it can be explained by residual confounding.

We did not observe a higher short-term stroke recurrence risk in our study, but this could be due to the limited number of patients with a cardiac thrombus. A previous study in non-stroke patients with a left ventricular thrombus did observe an approximate 10% risk of ischemic stroke during hospital admission.^20^ A recent study reporting on patients with stroke due to atrial fibrillation found that the recurrent stroke risk at 1-year follow-up was twice as high for patients with a cardiac thrombus identified on TEE compared to patients without a cardiac thrombus.^21^

The suggested long-term increase in stroke recurrence risk in patients with cardiac thrombi,^21^ combined with our data showing worse functional outcome, provides an argument to perform adequate cardiac imaging even in patients with a known cardioembolic source to identify those with a higher risk of poor outcome and recurrent stroke. Identification of a cardiac thrombus will not result in a change in long-term management in patients with a known cardioembolic source, but detecting a cardiac thrombus on cardiac CT during the acute stroke imaging protocol can aid clinicians to decide on continuation or early initiation of anticoagulant therapy. The optimal timing of anticoagulation therapy in patients with cardioembolic stroke is subject of various ongoing clinical trials.^22,23^ The AREST trial showed safety of early initiation of anticoagulation therapy in stroke patients with atrial fibrillation,^24^ and the TIMING trial demonstrated non-inferiority but not superiority of early initiation of anticoagulation in these patients.^25^ While the results of the remaining ongoing trials must be awaited, identification of cardiac thrombi may help to stratify patients into those most at risk for recurrent stroke, and thus those who may benefit most from early start of anticoagulation.^20^ However, more data are needed to establish whether patients with a cardiac thrombus benefit from early initiation of anticoagulation therapy, as they may have a higher short-term risk of stroke recurrence but may also be prone to hemorrhagic transformation due to large areas of cerebral infarction.

Previous studies showed that cardiac CT, which was not acquired during the stroke imaging protocol, has excellent sensitivity and specificity for the detection of cardiac thrombi compared to TEE, which is the reference standard.^26^ However, there is a lack of data comparing cardiac CT acquired during the acute stroke imaging protocol to TEE. These data are needed to determine whether cardiac CT during the acute stroke imaging protocol is not only superior to TTE for detection of cardiac thrombi, but is also suitable alternative for TEE. This may potentially lead to a reduction of TEE in the diagnostic work-up after stroke.

One of the limitations of cardiac CT during the stroke imaging protocol is that it requires advanced scanners, specialized software and trained personnel for acquiring and reporting cardiac CT in the acute phase of ischemic stroke. Moreover, cardiac CT results in additional radiation exposure. Future research is needed to determine if patient selection can be optimized to improve the yield of cardiac CT and reduce excess radiation in patients with ischemic stroke. Our data showed that patients with a cardiac thrombus have higher NIHSS scores at baseline. Larger datasets are needed to explore whether a NIHSS cut-off can be determined that can guide the decision to perform cardiac CT to screen for cardiac thrombi.

In total, 7/31 (23%) patients with a thrombus in the left atrial appendage did not have atrial fibrillation detected. The formation of left atrial appendage thrombi in these patients may be the result of atrial fibrillation despite ECG and rhythm-monitoring. It may be that loop-recorders would detect higher rates of atrial fibrillation in this group of patients compared to those patients without a cardiac thrombus. Alternatively, the formation of a left atrial appendage thrombus may be due to left atrial cardiopathy, which is increasingly recognized as source of cardio embolism. The idea of atrial cardiopathy is that ischemic stroke can originate from a thrombogenic atrial substrate even if there is no atrial fibrillation.^27^ Identified markers of atrial cardiopathy include the P-wave terminal force in lead V1 (PTFV1) on ECG, amino terminal pro-B-type natriuretic peptide (NT-proBNP) and left atrial enlargement. The ongoing ARCADIA trial aim to determine whether stroke recurrence in patient who have markers of atrial cardiopathy may be reduced by anticoagulation therapy rather than antiplatelet therapy.^27^

Approximately 40% of patients with a cardiac thrombus were treated with anticoagulation therapy prior to the index stroke. Development of a cardiac thrombus in these patients may be partially explained by subtherapeutic INR in patients treated with vitamin K antagonists which we observed in four out of nine patients in our study. For patients treated with a DOAC at baseline we assessed non-compliance and temporary discontinuation, but we do not have data on an objective marker (i.e. laboratory parameter) to determine whether there were subtherapeutic levels of DOAC at baseline in this group of patients.

Our study has several limitations. First, this was a single center study which limits the generalizability of the results. These findings should therefore be confirmed in other cohorts. Second, while this is one of the largest studies that examined cardiac thrombi in patients with acute ischemic stroke, the sample size is still limited. The study was especially underpowered to detect a difference in risk of recurrent stroke. Finally, we did not perform TEE in patients with cardiac thrombus to confirm the diagnosis. However, previous studies reported excellent sensitivity and specificity for the detection of cardiac thrombi when comparing cardiac CT with TEE.^26^

## Conclusion

Cardiac CT detected a cardiac thrombus in one in every 12 patients with acute ischemic stroke. Patients with cardiac thrombi more often had severe neurological deficits and multivessel occlusions. Presence of a cardiac thrombus was associated with worse functional outcome.
